# Bioprotection with *Saccharomyces cerevisiae*: A Promising Strategy

**DOI:** 10.3390/microorganisms13051163

**Published:** 2025-05-20

**Authors:** Fatima El Dana, Vanessa David, Raphaëlle Tourdot-Maréchal, Salem Hayar, Marie-Charlotte Colosio, Hervé Alexandre

**Affiliations:** 1 Laboratoire AFIM-IUVV, UMR Procédés Alimentaires et Microbiologiques, Institut Agro Dijon, INRAE, Université Bourgogne Europe, 21000 Dijon, France; fatima.eldana@gmail.com (F.E.D.); vanessa.vaizant@u-bourgogne.fr (V.D.); raphaele.tourdot-marechal@u-bourgogne.fr (R.T.-M.); 2 Research Platform for Environmental Sciences (EDST-PRASE), Doctoral School of Science and Technology, Lebanese University, Beirut 1003, Lebanon; hayarsalem@ul.edu.lb; 3 Faculty of Agronomy, Department of Plant Protection, Lebanese University, Dekwaneh 90775, Lebanon; 4 Institut Français de la Vigne et du Vin (IFV), 44120 Nantes, France; marie-charlotte.colosio@vignevin.com

**Keywords:** bioprotection, *Saccharomyces cerevisiae*, metabolites, toxins, yeast interaction, mechnaims of action

## Abstract

Bioprotection in winemaking refers to the use of naturally occurring microorganisms—mainly non-*Saccharomyces* yeasts—to inhibit the growth of spoilage microbes and reduce the need for chemical preservatives like sulfur dioxide (SO_2_). Numerous studies have demonstrated the benefits of non-*Saccharomyces* as bioprotectants. However, the use of *Saccharomyces cerevisiae* as a bioprotectant has been studied very little. Furthermore, it can offer many advantages for the production of sulfite-free wines. To test if *S. cerevisiae* could be used in bioprotection, we compared the ability of different strains to inhibit the growth of *Brettanomyces bruxellensis* and *Hanseniaspora uvarum*. Among the strains tested, the *S. cerevisiae* Sc54 strain isolated from the vineyard of the Bekaa plain was selected. To investigate its mechanisms of action, we analyzed its metabolite production, including acetic acid and ethanol. Taking into account the low levels of these metabolites and the lack of similar inhibition patterns in media supplemented with acetic acid and ethanol, it appears that other factors contribute to its antagonistic properties. Nutrient competition was ruled out as a factor, as the growth inhibition of *B. bruxellensis* and *H. uvarum* occurred rapidly within the first 24 h of co-culture. In this study, we explored the role of the *S. cerevisiae* killer toxin (Sc54Kt) as a bioprotective agent against *H. uvarum* and *B. bruxellensis* spoilage yeasts. Purification procedures with ethanol allowed the extraction of Sc54Kt, yielding two concentrations (0.185 and 0.5 mg/mL). Remarkably, semi-purified Sc54Kt exhibited inhibitory effects at both concentrations under winemaking conditions, effectively controlling the growth and metabolic activity of the target spoilage yeasts. Overall, these findings demonstrate that *S. cerevisiae* Sc54 not only exerts a strong bioprotective effect but also contributes to improving the quality of wine. The results suggest that *S. cerevisiae* Sc54 is a promising bioprotective agent for mitigating spoilage yeasts in winemaking, offering a natural and effective alternative to conventional antimicrobial strategies.

## 1. Introduction

Bioprotection in winemaking represents a significant shift in the approach to managing microbial populations during the fermentation process. This strategy primarily involves the use of non-*Saccharomyces* yeasts, which can inhibit spoilage organisms and enhance the overall quality of wine while reducing reliance on sulfur dioxide (SO_2_) as a preservative. The increasing demand for natural wines and consumer preference for products with fewer additives have encouraged research into bioprotective agents, particularly focusing on their efficacy and sensory impacts on wine. The concept of bioprotection is rooted in the deliberate inoculation of specific yeast strains that can outcompete undesirable microorganisms. For instance, species such as *Metschnikowia pulcherrima*, *Torulaspora delbrueckii*, and *Lachancea thermotolerans* have been identified as effective bioprotectants due to their ability to colonize grape musts rapidly and affect the growth of spoilage yeasts [1,2]. This competitive effect is crucial in the early stages of fermentation, where the risk of spoilage is highest. Studies have shown that these non-*Saccharomyces* yeasts can significantly reduce the incidence of off-flavors and enhance the aromatic profile of wines, thereby improving overall wine quality [3,4,5]. Research has demonstrated that bioprotective yeasts not only inhibit spoilage organisms but also contribute positively to the sensory attributes of the final product. For example, *M. pulcherrima* has been noted for its dual role as both a biocontrol agent and an enhancer of wine aroma [5]. The use of these yeasts can lead to an increase in fruity notes and a more complex aromatic profile, which is often desirable in both red and white wines [3,6]. Yeasts in wine also interact with lactic acid bacteria (LAB), and this relationship is shaped by factors like nutrient availability, oxygen, and ethanol levels [7]. Yeast metabolites, such as acetaldehyde, can inhibit or modulate LAB activity, while LAB may benefit from yeast-driven changes in the environment—such as oxygen reduction and the production of stabilizing compounds [8,9]. Additionally, the presence of *Oenococcus oeni* and its citrate metabolism can influence yeast activity and impact the development of wine aroma [7,10,11]. Furthermore, the application of bioprotective strategies has been shown to yield wines with distinct sensory characteristics when compared to those produced with traditional SO_2_ treatments, highlighting the potential for bioprotection to redefine wine profiles [3,12]. The effectiveness of bioprotection can vary as a function of several factors, including the specific yeast strains used, the winemaking techniques employed, and the characteristics of the grape must itself. For instance, the timing of inoculation and the conditions during fermentation can significantly influence the outcomes of bioprotective strategies [1,13]. In particular, the early addition of bioprotective strains during the prefermentation stage has been shown to be as effective as traditional sulfiting methods in preventing microbial spoilage [13]. This approach not only preserves the integrity of the wine but also in certain conditions could mitigate the potential for enzymatic and chemical oxidation, which can adversely affect wine quality [1,13]. Moreover, the integration of bioprotective yeasts into winemaking processes aligns with broader trends in the industry aimed at reducing chemical additives. Growing consumer awareness regarding the health implications of sulfites has prompted winemakers to explore alternative preservation methods [14,15]. Bioprotection offers a viable solution, allowing the production of wines that are both high in quality and lower in chemical additives, thus appealing to health-conscious consumers [6,15]. The application of bioprotection is not without its challenges. The variability in yeast performance across different vintages and grape varieties necessitates a tailored approach to bioprotective strategies [15,16]. Additionally, the potential for interactions between different yeast species during co-fermentation can lead to unpredictable outcomes, which require careful monitoring and management [16,17]. Nonetheless, the benefits of bioprotection, particularly in terms of reducing reliance on SO_2_ and enhancing wine quality, make it a compelling area of research and application within the wine industry. Recent studies have also explored the synergistic effects of combining bioprotective yeasts with other oenological practices, such as the use of oenological tannins and glutathione, to further enhance wine stability and sensory characteristics [18,19,20]. Bioprotection represents a promising avenue for improving wine quality and reducing the use of chemical additives in winemaking. The strategic use of non-*Saccharomyces* yeasts not only protects against spoilage but also enhances the sensory attributes of wine, aligning with consumer preferences for natural products. As we have seen, the use of *non-Saccharomyces* as a bioprotectant has several advantages. However, as explained above, the management of bioprotection is not always easy; moreover, it requires the use of a non-*Saccharomyces* followed by a *S. cerevisiae*, which is the only species that can complete alcoholic fermentation. In addition, the use of non-*Saccharomyces* as a bioprotectant represents an economic cost, as these strains are more expensive than *S. cerevisiae*. Surprisingly few studies have focused on the use of *S. cerevisiae* as a bioprotectant [21].

The use of *S. cerevisiae* as a bioprotective strain in winemaking could be very promising due to its ability to enhance fermentation processes while simultaneously mitigating the risks associated with spoilage microorganisms. One of the primary advantages of using *S. cerevisiae* lies in its ability to establish a competitive environment that can inhibit spoilage organisms [22]. For these reasons, we have investigated the use of *S. cerevisiae* as a bioprotective strain.

## 2. Materials and Methods

### 2.1. Microbial Strains, Storage, and Culture Media

Indigenous bioprotective *S. cerevisiae* strain Sc54 was isolated and identified during an isolation campaign of indigenous yeasts from grape berry in the Kefrayaa vineyard in Lebanon. All sensitive yeast strains belonging to *B. bruxellensis* coded B1, B250, B3 (NL6293), and B7 (NL6297) came from the Yeast Collection of the French Institute of Vine and Wine (Institut Français de la Vigne et du Vin (IFV), Nantes, France). *H. uvarum* Hu3137 was provided by the University Institute of Vine and Wine Jules Guyot (Institut de la Vigne et du Vin Université Bourgogne Europe (IUVV), Dijon, France). Three commercial *S. cerevisiae* yeasts, VL1, VL2, and X16 (LAFFORT/ZYMAFLORE^®^, Bordeaux, France), were used. *S. cerevisiae* S342 (NCYC 738) and S334 (NCYC 1006) were purchased (collection strains), and strain S340 (174 COEB) was provided by the COEB (Centre Oenologique de Bourgogne, Dijon, France). Commercial yeast strains were used as a reference to compare their killing activity against spoilage yeasts with that of the indigenous *S. cerevisiae* Sc54. This investigation allowed us to evaluate whether the bioprotective potential of Sc54 was comparable to or distinct from widely used commercial starters.

Pre-cultures of all yeast strains were grown in 40 mL of YPD broth (10 g/L yeast extract, 20 g/L peptone, 20 g/L glucose) supplemented with 0.2 g/L chloramphenicol (Sigma-Aldrich, Saint-Quantin-Fallavier, France) and buffered to pH 3.5 using 0.1 M hydrochloric acid/citric acid. The cultures were incubated overnight at 22 °C, except for *B. bruxellensis* strains, which required an extended incubation of 72 h due to their slower growth rate. All yeast strains were stored at −80 °C in a cryoprotectant mixture of 50% glycerol and 50% YPD broth. The killing activity of *S. cerevisiae* against *B. bruxellensis* and *H. uvarum* was assessed on YPD-MB agar on plates at pH 3.5, which consisted of YPD medium supplemented with 0.03 g/L methylene blue and 20 g/L agar, and in Synthetic used in our previous studies.

### 2.2. Molecular Profiling and Identification of S. cerevisiae Strains

#### 2.2.1. Genomic DNA Extraction

DNA was extracted from isolated *S. cerevisiae* colonies following a standard cell lysis and precipitation method. Individual yeast colonies were transferred into 1.5 mL microcentrifuge tubes (NEST Biotechnology, Jiangsu, China) containing 660 µL of 50TE buffer supplemented with SDS. The lysis buffer contained Tris-EDTA (Eurobio Scientific, Les Ulis Cedex, France) and sodium dodecyl sulfate (SDS; Sigma-Aldrich, St. Louis, MO, USA) to lyse cells. Samples were mixed well by vortexing, and then incubated at 65 °C for 30 min (Heraeus incubator, Hanau, Germany) [23,24,25].

After lysis, 340 µL of 5 M potassium acetate (KAc; Sigma-Aldrich, St. Louis, MO, USA) was added to each tube to precipitate cellular debris. Tubes were then incubated at 4 °C for 30 min and centrifuged at 13,000 rpm for 10 min. The supernatant (~750 µL) (which contained the DNA) was gently pipetted off to new microcentrifuge tubes [23].

The DNA was then precipitated with an equal volume (750 µL) of isopropanol with gentle mixing, and allowed to sit at room temperature for 10 min. Samples were subsequently centrifuged at 13,000 rpm for an additional 10 min to pellet the DNA. The supernatant was removed, and the DNA pellet was washed with cold 95% ethanol to eliminate the remaining isopropanol. After a quick centrifugation and removal of ethanol, pellets were air-dried for 20 min in 250 µL of TE buffer. The purified DNA was kept at −20 °C for additional molecular study [23,24,26,27].

#### 2.2.2. InterDelta-PCR Fingerprinting

InteDelta PCR was applied to amplify the δ (TY1 retrotransposon) region with the purpose of identifying isolates of the same species *S. cerevisiae*, in particular, those obtained from the first 12 grape varieties [28]. Amplification was carried out using Δ Mix Taq polymerase (1220 μL H_2_O, 160 μL Dilution 10× + MgCl_2_, 64 μL DNTP (mix ATGC), 40 μL MgCl_2_) with the δ12 (5′-TCAACAATGGAATCCCAAC-3′) and δ21 (5′-CATCTTAACACCGTATATGA-3′) primers, as described by EL Dana et al. [23] and De Celis et al. [28]. PCR reactions were prepared in PCR tubes (SSI Bio, Lodi, CA, USA) and performed in a SimpliAmp thermal cycler (Thermo Fisher Scientific, Singapore). The amplification program was an initial 5 min denaturation at 95 °C and then 40 cycles of 30 s at 95 °C, 1 min at 46 °C, and 1 min at 72 °C. The final extension was carried out at 72 °C for 5 min [23,24,27,28,29].

Restriction fragments were separated by electrophoresis on a 1.5% agarose gel (Sigma-Aldrich, Chemie GmbH, Munich/Schnelldorf, Germany) prepared using a 1:1 mixture of standard agarose and resophor agarose in 100 mL of 1× TBE buffer containing 5% Midorigreen DNA stain (NIPPON Genetics EUROPE, Tokyo, Japan). A 100 bp DNA ladder was included as a molecular size marker. Electrophoresis was performed to allow DNA migration, and the resulting bands were visualized under UV light using the E-box VX2/20MX imaging system (VILBER, Marne-la-Vallée Cedex 3, France). DNA banding patterns were compared to the molecular marker to distinguish genetically distinct yeast strains [23,30].

### 2.3. Killing Acticity Assay and Measurment

The killing activity of *S. cerevisiae* strains was evaluated using a diffusion plate assay against a panel of yeasts, including *H. uvarum* Hu3137 and *B. bruxellensis* strains B1 and B250. The plates were seeded with potential sensitive strains at a final concentration of 10^6^ CFU/mL on YPD agar (10 g/L yeast extract, 20 g/L peptone, 20 g/L glucose, 18 g/L agar, with the addition of 0.2 g/L of chloramphenicol (Sigma-Aldrich, Saint-Quantin-Fallavier, France), pH 3.5. An equal concentration (10^6^ CFU/mL) of bioprotectant *S. cerevisiae* strain was then spotted onto the plates. After 72 h of incubation at 22 °C, a strain was classified as a killer yeast if its colony was surrounded by a clear inhibition zone. The assay was performed in triplicate, and the inhibition zone diameter was measured using a caliper. Killing activity (KA) was expressed in arbitrary units (AU) per mL (diameter of the inhibition zone in mm), where 1 AU corresponded to the amount of toxin required to produce a 13 mm inhibition zone.

### 2.4. Antagonism of S. cerevisiae Against Spoilage Yeast in Synthetic Must

Single and mixed cultures were carried out using the *S. cerevisiae* strain Sc54, along with *H. uvarum* Hu3137 and *B. bruxellensis* strains B1 and B250. The fermentations were conducted in Erlenmeyer flasks sealed with carded cotton, each containing 100 mL of sterile synthetic must SM300 (SM), formulated based on Bely et al. [31] and further described by Evers et al. [32]. Mixed cultures were conducted simultaneously using the *S. cerevisiae* strain at an initial cell density of 10^6^ CFU/mL, alongside *B. bruxellensis* and *H. uvarum* strains at 10^4^ CFU/mL each, simulating low contamination levels typically observed on grapes at advanced maturity (possibly reaching 10^6^ CFU/mL) [33]. Additionally, two single cultures were performed with *H. uvarum* (Hu3137) and *B. bruxellensis* (B1 and B250) at an initial density of 10^4^ CFU/mL, serving as negative controls to assess the antagonistic effect of the above-mentioned *S. cerevisiae* strain Sc54. A single culture of Sc54 (10^6^ CFU/mL) was also carried out to evaluate its standard growth in the absence of spoilage yeasts.

All fermentations were performed at 22 °C in triplicate. From each flask, 500 μL of culture (single and mixed cultures) was aseptically sampled at 0, 1, 2, 3, 6, 7, and 9 days following the inoculation of the strains. Of this, 100 μL was allocated for cell enumeration, while the remaining 400 μL was stored at −20 °C for subsequent chemical analyses.

### 2.5. Measurement of Yeast Growth

Fermentation samples were collected to monitor yeast growth dynamics. The culturability (CFU/mL) of *S. cerevisiae*, *B. bruxellensis*, and *H. uvarum* was assessed using the conventional plating method. Total yeast and viable *S. cerevisiae* cells were enumerated on YPD agar medium. To differentiate colony counts of *B. bruxellensis* and *H. uvarum* from *S. cerevisiae* in mixed culture samples, a selective ITV medium (20 g/L glucose, 10 g/L yeast extract, 20 g/L tryptone, 0.1 g/L para-coumaric acid, 0.1 g/L ferulic acid, 0.03 g/L bromocresol green, 0.2 g/L chloramphenicol, and 20 g/L agar, pH 3.5, supplemented with 0.006% (*v*/*v*) cycloheximide) was used, following the procedure described by Branco et al. [34] to determine the cell concentration of *B. bruxellensis* and *H. uvarum* in the mixed-culture fermentations. The cell count was carried out after 48 h incubation at 22 °C.

### 2.6. Killer Toxin Production and Quantification

To establish the highest amount of toxin production, *S. cerevisiae* Sc54 was cultivated in 300 mL of synthetic must at pH 3.5. The culture was agitated at 150 rpm on a rotatory shaker at 22 °C. After 24 h, the cells were discarded after centrifugation at 4100 rpm for 30 min at 4 °C. The protein fraction containing Sc54Kt was obtained by precipitating the culture supernatant with 96% ice-cold ethanol (2:1 *v*/*v*) for 24 h at +4 °C, followed by centrifugation for 20 min at 12,000 rpm [35,36,37]. The resultant pellet was resuspended in a 0.1 M sodium phosphate buffer, pH 7.0. This supernatant was used as the killer toxin extract [35,36]. Aliquots of supernatants were either used immediately or stored at 4 °C for further analysis of killing activity.

Killer toxin quantification was performed using the dye binding method of the Bradford protein assay (Bradford, 1976) [38,39,40,41]. The protein was stained with 1× Bradford reagent (Bio-Rad, Hercules, CA, USA), and the protein concentration determined by comparison with seven known concentrations of proteins (bovine serum albumin (BSA)). The concentration of the semi-purified killer toxin was determined at 595 nm by UV-Visible light spectrophotometer (Genesys 50, Thermo Fisher Scientific Inc., Waltham, MA, USA). Two concentrations of Sc54Kt, 0.185 and 0.500 mg/mL, were adopted during the killing activity monitoring on agar in SM.

### 2.7. Well Plate Assay of Sc54Kt and Arbitrary Unit

In order to determine possible effects of purified toxin on spoilage yeasts, killing activity (KA) tests were carried out in the laboratory of IUVV. After incubation of *B. bruxellensis* (B1 and B250) and *H. uvarum* (Hu3137) at an initial concentration of 10^4^ CFU/mL on YPD-MB agar plates at pH 3.5, semi-purified Sc54Kt at two different concentrations, 0.185 and 0.500 mg/mL was added into 5 mm wells in triplicate. The Sc54Kt toxin was allowed to interact with the spoilage cells for 24–48 h at 22 °C. One arbitrary unit (AU) defined as the toxin concentration caused a clear zone of 1 mm, as previously described [42,43]. After 24–48 h of incubation at 22 °C, the inhibition halos generated around each well were measured, and the values obtained indicated the killing effect at each concentration.

### 2.8. Sensitivity of B. bruxellensis and H. uvarum Strains to Sc54Kt Toxin in Must

To evaluate the sensitivity of *B. bruxellensis* (B1 and B250) and *H. uvarum* (Hu3137) to the Sc54Kt toxin in must, these yeast strains were cultured in the presence of varying concentrations of the bioactive peptidic fraction, purified as described in the Section 2.6. Growth inhibition assays were conducted in Erlenmeyer flasks, with three independent replicates. Each flask contained 100 mL of synthetic must SM300 (pH 3.5) either without Sc54Kt (negative control) or supplemented with the toxin at a final protein concentration of 0.185 or 0.500 mg/mL. The media were inoculated with 10^4^ CFU/mL of each of the above-mentioned B1, B250, and Hu3137 strains, and the flasks were incubated at 22 °C. Cell growth was followed during time intervals of 0, 5, 24, and 72 h by plate enumeration on YPD agar medium.

### 2.9. Analytical Determinations

Yeast chemical compound production was quantified in synthetic must (SM). Cultures were conducted in three biological replicates at 22 °C in 100 mL glass bottles, each containing 100 mL of SM, and inoculated with each of Sc54, B1, B250, and Hu3137 as monoculture and in co-culture. The concentrations of ethanol and acetic acid were determined using an automated Y15 analyzer (BioSystem, Muttenz, Switzerland) and FT-IR spectroscopy with the OenoFoss™ analyzer (Ecoparc de Nanterre, Nanterre, France). Following centrifugation at 7000 rpm for 10 min, 200 μL of each sample was analyzed according to the supplier’s instructions.

### 2.10. Statistical Analysis

Growth parameters and killer toxin activity were evaluated using one-way ANOVA, with Levene’s test applied to verify variance homogeneity. Welch’s *t*-test was used for pairwise comparisons when variances were unequal, with a significance threshold of α = 0.05. Metabolite production and relative intensities were analyzed using Welch’s *t*-test for two-group comparisons (*n* = 2) and ANOVA followed by Tukey’s post hoc test for comparisons involving more than two groups (*n* = 3), maintaining a 5% significance level. Cell growth assessment, maximum growth rate (µmax h^−1^), graphical analyses, and additional statistical evaluations were performed using IMB SPSS Statistics (version 22).

## 3. Results and Discussion

Five strains of *S. cerevisiae*, including one indigenous strain isolated from Lebanese grape must, were selected to evaluate the diversity of their antagonistic effects within the species under oenological conditions. This study further aimed to evaluate the inhibitory effects of selected *S. cerevisiae* strains in synthetic must (SM) and to elucidate the mechanisms underlying their antimicrobial action. Their antimicrobial activities were tested against two spoilage microorganisms: *H. uvarum*, known for producing ethyl acetate and acetic acid, and *B. bruxellensis*, which is responsible for phenolic off-flavors that detrimentally affect the sensory properties of wine [44,45].

### 3.1. Evaluation of Killing Activity

The killing efficacy of a panel of *S. cerevisiae* yeasts, Sc54, S342, S340, S334, and VL2, was evaluated against the wine spoilage yeasts *B. bruxellensis* and *H. uvarum.* The results demonstrated that under the condition tested (YPD—MB agar, pH 3.5 at 22 °C), Sc54 significantly inhibited the growth of all these microbial species, exhibiting inhibition zones exceeding 13 mm (Table 1). However, the other *S. cerevisiae* strains displayed varying levels of killing activity, with strong to apparent effects against B1, and apparent to mild effects against B250 and Hu3137. The current findings demonstrated that various *S. cerevisiae* strains exhibit activity against a broad range of microorganisms that cause wine spoilage. Nevertheless, the response of these microbial species was strain/species specific, as evidenced by the differential sensitivity shown by various strains of *B. bruxellensis* and *H. uvarum*, detailed in Table 1.

With an initial *H. uvarum and B. bruxellensis* concentration of 10^6^ CFU/mL, strains Sc54, S342, S340, S334, and VL2 displayed an inhibitory effect, though their efficacy varied. Indeed, strain Sc54 showed the most robust and consistent antimicrobial activity against both spoilage yeasts *B. bruxellensis* (B1 and B250) and *H. uvarum* (Hu3137), positioning it as a good candidate for bioprotective assays against these spoilage organisms. Similarly, Li et al. [46] investigated the killing activity of two *S. cerevisiae* strains (RV002 and RV171), selected from the Yun-Nan Hong vineyard (China), against *H. uvarum* using YEPD-MB agar. However, their study employed a three-log difference in cell density between the strains (10^8^ and 10^5^ CFU/mL, respectively) rather than a comparable population density as used in our work. Notably, only *S. cerevisiae* RV002 exhibited an inhibitory effect, forming a clear halo zone against *H. uvarum*, whereas RV171 had no observable impact.

While direct studies on the cell–cell interactions between *S. cerevisiae* and *B. bruxellensis* are limited, research on related yeast species provides valuable insights. A study by Hu et al. [47] investigated the effects of cell–cell contact between *Pichia kluyveri* and *S. cerevisiae* during alcoholic fermentations. The findings revealed that such interactions led to decreased cell viability for both yeasts, increased production of acetate and ethyl esters, and altered amino acid consumption patterns, notably affecting glutamic acid and specific amino acids linked to cell growth. Branco et al. [48] explored the use of saccharomycin-overproducing *S. cerevisiae* strains to control *B. bruxellensis* in alcoholic fermentations. Their study demonstrated that these engineered strains effectively inhibited the growth of *B. bruxellensis*, suggesting a potential bioprotectant approach in winemaking.

To test the bioprotective potential of the selected strain, the Sc54 strain was chosen for further exploration in synthetic must due to its pronounced inhibitory characteristics.

### 3.2. Biopreservative Efficacy of Sc54 in Co-Culture with B. bruxellensis and H. uvarum in SM

**Figure 1:**
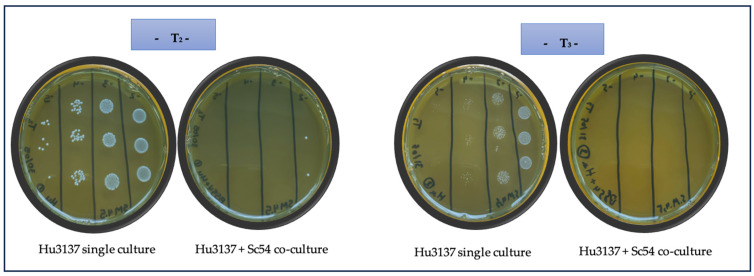
The effectiveness of the *S. cerevisiae* Sc54 strain on *H. uvarum* in co-culture within synthetic must (pH 3.5) was assessed at 22 °C after 2 (T2) and 3 (T3) days of inoculation. Cell viability was determined through viable plate counts. A single culture of Hu3137 served as control, while co-cultures with Sc54 were evaluated. All strains and conditions were assessed using identical dilution series (10^−2^, 10^−3^, 10^−4^, 10^−5^). The data represent three technical replicates derived from three independent experiments.

**Figure 2:**
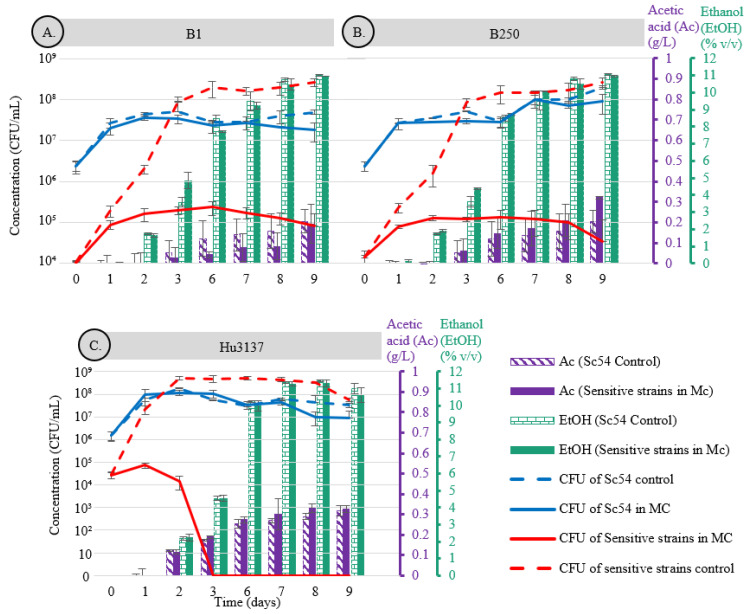
Growth curves of *B. bruxellensis* (B1, B250) *and H. uvarum* (Hu3137) in single culture (dotted red line) and co-culture (solid red line) with *S. cerevisiae* Sc54 at 22 °C. Panels (**A**–**C**) illustrate the growth profiles of spoilage yeast strains at initial inoculum levels of 10^4^ and 10^6^ CFU/mL over a 9-day fermentation period. The growth behavior of Sc54 in single culture (dotted blue line) and co-culture (solid blue line) is also presented. Metabolite production, including acetic acid 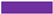 and 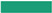 ethanol, is displayed as striped bars for Sc54 in single culture and solid bars for co-culture conditions.

Yeast growth profiles and metabolite production during fermentations in synthetic must (SM) at pH 3.5, conducted with *B. bruxellensis* (strains B1 and B250) and *H. uvarum* (strain Hu3137) in single and mixed cultures with *S. cerevisiae* Sc54, are illustrated in Figure 1 and Figure 2. During co-fermentations (Figure 2), the population of *S. cerevisiae* increased from 4 × 10^6^ CFU/mL to 5.10 × 10^7^ CFU/mL within 3 days, maintaining this level with minor fluctuations until the end of fermentation (day 9). Neither *B. bruxellensis* strains B1 and B250 nor *H. uvarum* Hu3137 exhibited any inhibitory effects on *S. cerevisiae* growth. In contrast, both *B. bruxellensis* strains experienced a rapid decline in cell density during the first 2 days when incubated with *S. cerevisiae* Sc54, decreasing to below 1.61 × 10^5^ and 1.29 × 10^5^ CFU/mL, respectively, attaining more than 1 log difference with that of growth in the control medium (Figure 2A,B). This reduction continued, with populations stabilizing at approximately 10^5^ CFU/mL for 7 days, before further decreasing to 8.10 × 10^4^ and 3.33 × 10^4^ CFU/mL by day-9, significantly lower than the control cultures, which maintained about 3 × 10^8^ CFU/mL. The growth rate of B1 and B250 was negatively impacted by the interaction with Sc54 after 3 days of inoculation (*p* < 0.05), in terms of a slowdown in μmax values, of 0.042 ± 0.003 and 0.026 ± 0.01 h^−1^, respectively (Table 2), compared to that in single culture with values of 0.126 ± 0.001 and 0.123 ± 0.002 h^−1^, respectively.

*S. cerevisiae* Sc54 demonstrated a pronounced and rapid killing effect on *H. uvarum* Hu3137. The population density of Hu3137 cells decreased to 1.47 × 10^4^ CFU/mL by day 2, but then, *H. uvarum* cells died off directly by the 3rd day of incubation, with no visible culturability throughout the entire fermentation (>1 CFU/mL at day 9) (Figure 2C). Conversely, during the single-culture fermentation, the *H. uvarum* population increased from an initial count of 10^4^ CFU/mL at day 0 up to 5.08 × 10^8^ CFU/mL by day 2, stabilizing around 6 × 10^7^ CFU/mL through day 9 (Figure 2C). Similar results were reported by Li et al. [46]. Furthermore, in co-culture with Sc54, the *H. uvarum* population exhibited a much lower growth kinetics μmax value (−0.01 ± 0.012 h^−1^) than in single culture (0.204 ± 0.001 h^−1^) for the first 3 days (*p* > 0.05) (Table 2).

The mechanism causing the premature death of *H. uvarum* and inhibition of *B. bruxellensis* in the presence of *S. cerevisiae* in synthetic must could be attributed to various interactions. Metabolic profiles (i.e., acetic acid and ethanol production) during the mixed-culture fermentation of *S. cerevisiae*/*B. bruxellensis* and *S. cerevisiae*/*H. uvarum* (Figure 2) showed high ethanol production levels, attaining a maximal concentration of approximately 11% (*v*/*v*), and a low level of acetic acid production not exceeding 0.3 g/L at day 9 for all co-mixtures. Indeed, it is recognized that these metabolites can inhibit cell growth. However, the amount of acetic acid present during the inhibition of Hu3137, B1, and B250 could not trigger the latter’s death (0.3–0.4 g/L acetic acid). Although this could be attributed to high ethanol production, this theory was dismissed. Indeed, by the time *B. bruxellensis* and *H. uvarum* inhibition occurred on the first day, ethanol had not yet been produced, though on the second day, a significant decrease in the population growth of these yeasts strains was further observed, reaching about 10^5^ CFU/mL and 10^4^ CFU/mL for *B. bruxellensis* (B1 and B250) and *H. uvarum* (Hu3137), respectively, at the time when the ethanol level did not exceed 2–3% *v*/*v*, as shown in Figure 2. These ethanol levels were not capable of causing such a significant decline in *B. bruxellensis* and *H. uvarum* cell populations. Indeed, we confirmed that ethanol up to 8% (*v*/*v*) in synthetic must only exerted a mild effect not exceeding 0.5 log difference on both species (supplementary data). Additionally, both *H. uvarum* and *B. bruxellensis* are known to have a high tolerance for ethanol, reportedly up to 9–12% *v*/*v* and 14% *v*/*v*, respectively, as previously noted [49,50,51]. Another hypothesis could be nutrient competition, but this theory can be discounted. The medium was rich enough to maintain up to 5 × 10^8^ CFU/mL for *H. uvarum* and 10^8^ CFU/mL for *B. bruxellensis* in single cultures, after 2 days. However, in co-culture, the population peaked at only 10^5^ CFU/mL, considerably less than the potential maximum over the same time frame. Given that the same medium allowed for exponential growth in monocultures over the same time frame, it is unlikely that nutrient depletion occurred early enough to limit growth in the co-cultures.

In winemaking, bioprotective strains are often introduced during the pre-fermentation phase [45]. Consequently, this study focused on examining the interaction between *S. cerevisiae* and spoilage yeasts on the first days after inoculation, a critical point for observing microbial dynamics. By this time, a significant reduction in the populations of *B. bruxellensis* and *H. uvarum* was noted, indicating the effective inhibition of these spoilage yeasts by *S. cerevisiae*. Our results indicate that *S. cerevisiae* Sc54 exerted an antagonistic effect against these spoilage yeasts under oenological conditions, demonstrating a significant bioprotective capability. These findings led us to hypothesize that the early cell death and growth inhibition observed may be attributed to the production of killer toxins, suggesting a potential mechanism of action by which *S. cerevisiae* Sc54 protects against these spoilage yeasts. To our knowledge, this is the first report on the use of *S. cerevisiae* as a bioprotective agent in winemaking conditions against *B. bruxellensis* and *H. uvarum*. However, Kemsawasd et al. [52] reported the ability of *S. cerevisiae* to inhibit *L. thermotolerans* in YPG modified medium. According to their results, the death of *L. thermotolerans* in mixed cultures with *S. cerevisiae* was caused by a combination of cell-to-cell contact and antimicrobial peptides.

### 3.3. Effect of Killer Toxin Sc54Kt on B. bruxellensis and H. uvarum Culturability and Growth

#### 3.3.1. Killing Activity Assay of Sc54Kt

After partial purification with ethanol, the killing activity in the supernatant separated from the cells was analyzed. The evaluation of the killing activity of Sc54Kt was the first step toward the practical application of this killer toxin in the control of spoilage (*B. bruxellensis*) and apiculate (*H. uvarum*) yeasts in winemaking. The results obtained after well test assays indicated that upon treatment with semi-purified Sc54Kt toxin, the growth of *B. bruxellensis* (B1 and B250) and *H. uvarum* (Hu3137) was inhibited (Table 3). *H. uvarum* proved to be the most sensitive yeast, with a wider inhibition halo observed, followed by B1 and then B250 of *B. bruxellensis*. The addition of 0.5 mg/mL of Sc54Kt revealed an increase of 4–6 mm in the inhibition halo against *B. bruxellensis* and *H. uvarum* if compared with the lower concentration, 0.185 mg/mL, as illustrated in Table 3, confirming the toxin’s dose-dependent efficacy.

The present results revealed that Sc54Kt was active against two varieties of microorganisms associated with wine fermentation, although the sensitivity of these microbial species towards the killer toxin was strain- and species-specific, as evidenced by differing responses among different strains of *B. bruxellensis* and by that of *H. uvarum.* However, it is important to note that the inhibitory activity of the toxins can vary significantly between solid agar and liquid synthetic must environments. This variation prompted further investigation into the potential of Sc54Kt in SM within this study, highlighting the need to evaluate toxin activity under conditions that closely mimic winemaking processes.

Albergaria et al. [53] provided a comprehensive review discussing the inhibition of *B. bruxellensis* growth by non-*Saccharomyces* yeasts. However, the killing activity of killer toxins naturally extracted and tested under real oenological conditions against *B. bruxellensis* appeared to be quite limited. Among these experiments, some trails were performed in YEPD medium, which does not accurately mimic real winemaking conditions, as demonstrated in [34]. Moreover, another study employed a very low initial concentration of *B. bruxellensis*, 5 × 10^2^ CFU/mL, and other findings revealed that *B. bruxellensis* growth inhibition in both wine types was only achieved when 1.0 mg/mL of saccharomycin was added, in addition to being supplemented with SO_2_ at concentrations of 25 and 50 mg/mL PMB [48,53,54]. None of these studies represent the optimal conditions and concept of real bioprotection. In summary, these studies demonstrated that the minimal inhibitory concentration (MIC) required to inhibit six *B. bruxellensis* strains (*ISA 1649*, *ISA 1700*, *ISA 1791*, *ISA 2104*, *ISA 2116*, and *ISA 2211*) ranges between 1 and 2 mg/mL [34]. This is considerably higher than the concentrations used in our study (0.185 and 0.5 mg/mL), highlighting the increased effectiveness of our approach. On the other hand, as far as we know, no studies have directly examined the effect of killer toxins extracted from *S. cerevisiae* on *H. uvarum*. Existing research has only focused on cell–cell interactions and the effect on wine quality during mixed fermentations involving these two yeast species [46,55,56,57].

#### 3.3.2. Spectrum of Action and Antimicrobial Properties of Killer Toxin Sc54Kt in Synthetic Must

**Figure 3:**
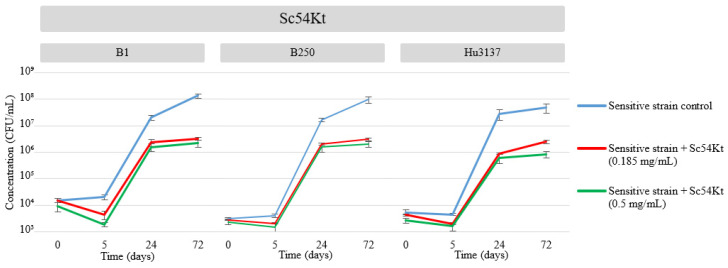
Growth curves of *B. bruxellensis* and *H. uvarum* and the killing activity of Sc54Kt were monitored during microfermentations. Growth curves (left axis): blue solid line represents the control without Sc54Kt; red solid line indicates addition of 0.185 mg/mL Sc54Kt; green solid line indicates addition of 0.5 mg/mL Sc54Kt. The curves demonstrate killing activity at 0, 5, 24, and 72 h for both concentrations. Data represent the mean of three independent experiments, each performed in triplicate.

In order to further confirm the involvement of natural killer toxin Sc54Kt in inhibiting the growth of *B. bruxellensis* and *H. uvarum* in wine, a synthetic must medium (SM300) mimicking the composition of wine (pH 3.5) was supplemented with two concentrations (0.185 and 0.5 mg/mL) of the killer toxin containing the natural biocide and artificially contaminated with 10^4^ CFU/mL of *B. bruxellensis* (B1 and B250) and *H. uvarum* (Hu3137). Yeast cell counts were monitored at intervals of 0, 5, 24, and 72 h after inoculation at 22 °C. Growth kinetics and culturability (CFU/mL) profiles for strains B1, B250, and Hu3137 in both the Sc54Kt biocide assay and the control assay (without biocide) are depicted in Figure 3.

The results indicated that in the control assay, *B. bruxellensis* B1 and B250 were able to proliferate normally to 1.70 × 10^7^ and 1.04 × 10^7^ CFU/mL, respectively, after 24 h of inoculation. In the biocide assay, when exposed to 0.5 mg/mL of Sc54Kt, the culturability of B1 and B250 dropped to 1.59 × 10^6^ and 2.29 × 10^6^ CFU/mL, respectively, as shown in Figure 3. This reduction remained stable upon addition of Sc54Kt with a difference of almost 2-log compared to the controls, where B1 and B250 culturability increased continuously, attaining a cell density of 10^8^ CFU/mL at 72 h. A slightly different phenomenon was observed upon exposure to 0.185 mg/mL of Sc54Kt, where a less pronounced effect was observed, as depicted by the second profile (green solid line).

A similar behavior was observed with Hu3137, albeit more markedly, where *H. uvarum* exhibited significantly higher concentrations in control (3 × 10^7^ CFU/mL) compared to the culture with Sc54Kt concentrations of 0.5 mg/mL and 0.185 mg/mL (6 × 10^5^ CFU/mL and 8.8 × 10^5^ CFU/mL, respectively) within 24 h of inoculation, presenting nearly a 2-log difference. Without toxin addition, *H. uvarum* culturability remained steady, achieving maximum growth kinetics of 5 × 10^7^ CFU/mL at 72 h of culture. However, in the presence of 0.5 and 0.185 mg/mL of Sc54Kt, the maximum cell densities observed were 8.43 × 10^5^ and 2.5 × 10^6^ CFU/mL, respectively (Figure 3).

The results from adding the killer toxin to the fermentation medium did not align precisely with observations from killer strain × sensitive strain co-culture experiments (Section 3.2), despite the toxin concentrations being theoretically equivalent to the bioprotectant levels in co-culture. This discrepancy could be due to higher toxin levels in co-culture than what is achievable in isolation, as the extraction and purification processes do not guarantee 100% yield of the toxins. Although the toxins used were in limited quantities, they still effectively inhibited the growth of spoilage yeasts. These data highlight that killer toxins likely represent the primary mechanism through which *S. cerevisiae* exerts its killing effects against spoilage yeasts, reinforcing the potential of bioprotective strategies in winemaking. In addition, they prove the killer toxin’s dose-dependent effect on both *B. bruxellensis* and *H. uvarum* inhibition.

Previous studies have demonstrated that killer toxin produced by *S. cerevisiae* possesses antimicrobial activity [34,48,58]. A minimum killer toxin concentration ranging from 1 to 2 mg/mL is required to inhibit *B. bruxellensis* [34,48,58], which is approximately 10-fold over the levels typically found in *S. cerevisiae* fermentation supernatants [14,15]. However, several tests of saccharomycin killing potentiality were conducted in YEPD medium [34,48,58,59], which does not replicate the composition of wine, thus providing results that may not reliably translate to natural environments. Concurrently, it has been demonstrated by Branco et al. [54] that 1 mg/mL of toxin alone is insufficient, even with an initial *B. bruxellensis* population as low as 10^2^ CFU/mL. The authors showed that the effective inhibition of *B. bruxellensis* growth in wine required the addition of 1.0 mg/mL of saccharomycin along with SO_2_, utilizing both 25 and 50 mg/mL of potassium metabisulfite. Concerning the studies on *H. uvarum*’s response to saccharomycin, research is limited compared to *Hanseniaspora guilliermondii*, which is inhibited at concentrations above 0.25 mg/mL) [34,58,60]. However, saccharomycin is not the only killer toxin produced by *S. cerevisiae*, so the observed inhibition might be due to another killer toxin. However, previous studies have demonstrated that classical killer toxins like K1, K2, and K28 from *S. cerevisiae* are only effective within their own species [60]. It was [61] found these toxins did not cause early death in *H. guilliermondii*. In addition, genetically modified *S. cerevisiae* strains were developed to overproduce these GAPDH-derived antimicrobial peptides (AMPs) to exert higher efficiency [48].

Understanding the mode of action of killer toxins is essential for optimizing their use as biocontrol agents and for ensuring specificity against target spoilage yeasts. Investigating the mechanistic details (e.g., membrane disruption, receptor binding, or enzymatic digestion of cytoskeletal structures) is also important, as it can shed light on the inhibitory effectiveness of antimicrobial agents. Few studies have investigated the mechanisms of death induced by killer toxins from *S. cerevisiae.* However, new evidence has begun to unravel the complex functioning of killer toxins. Another study [58] demonstrated that the synthetic antimicrobial peptide (AMP), saccharomycin—secreted by *S. cerevisiae* CCMI 885—exerted membrane-disrupting effects, induced apoptotic molecular markers, and was internalized in sensitive yeast species *H. guilliermondii* and *B. bruxellensis*. Similarly, Villalba et al. [59] reported that the killer toxin SeKT, produced by *Saccharomyces eubayanus*, acts on spoilage yeasts, including *B. bruxellensis, P. membranifaciens, M. guilliermondii*, and *P. manshurica*, by disrupting the cell wall through enzymatic β-glucanase and chitinase activities, leading to both necrotic and apoptotic cell death in a dose-dependent manner. A comparable mechanism was described [62], that Kpkt, a killer toxin secreted by *T. phaffii*, compromises cell wall integrity via a highly specific β-glucanase activity.

*S. cerevisiae* produces multiple known killer toxins, including K1, K2, K28, and Klus, which are the most known and characterized, with each using different mechanisms of action. K1 is the most characterized and is known to exert two sequential actions: firstly, the binding of the sensitive yeast cell wall 1,6-β-D-glucan receptor, and secondly, destabilization of the plasma membrane [63,64,65,66]. This disruption leads to the efflux of potassium ions and ultimately results in cell death. An alternative hypothesis is that K1 induces the formation of pores in the membrane that result in the uncontrolled dissipation of intracellular molecules such as protons, ATP, and ions [67,68]. These results demonstrate that K1’s cytotoxic action is mostly due to membrane action, rather than cell wall activity. K2 also demonstrates a two-phase killing mechanism, similarly to K1 and K28, with the same initial binding as K1 but through 1, 6-β-D-glucan-based receptor and Kre1p protein [69,70,71,72]. However, the subsequent cellular responses differ between K1 and K2, which allows strains producing one toxin to kill strains producing the other—despite being immune to their own toxin. K28 functions appear completely different, inhibiting the target cell cycle rather than the cell wall or membrane. This makes its molecular action more complex compared to K1 and K2 [73,74]. The Klus toxin is the most recently identified among *S. cerevisiae* killer toxins. The precise mechanism remains unknown, and it kills strains that produce K1, K2, and K28, while being immune to its own lysis [75,76]. Killer toxin Klus is relatively less aggressive towards sensitive cells as compared to killer toxins from other systems.

As far as we know, this work is the first well-established experiment demonstrating a killer toxin effect against *H. uvarum* in synthetic must. Indeed, in our study, Sc54Kt effectively inhibited the growth of both *B. bruxellensis* and *H. uvarum* at an initial population of 10^4^ CFU/mL, achieved with killer toxin concentrations as low as 0.5 mg/mL and even 0.185 mg/mL without conjugation with additional additives such as SO_2_, in synthetic must (pH 3.5, 22 °C), replicating authentic winemaking conditions.

## 4. Conclusions

This study showed the ability of *S. cerevisiae* to limit the development of spoilage yeast in oenological conditions during a fermentation. These properties make it possible to consider the use of this strain as a bioprotectant. Indeed, to our knowledge, few scientific studies have focused on *S. cerevisiae* species for bioprotection. Here, we presented a rare study regarding the use of *S. cerevisiae* as a bioprotective yeast strain. Our results demonstrated that the selected *S. cerevisiae* strain was able to limit the development of spoilage yeast and thus could be useful as an alternative to sulfite. Moreover, this strain possesses favorable traits for applications requiring fermentation performance. The use of *S. cerevisiae* as a must bioprotection yeast offers several other advantages over non-*Saccharomyces* yeasts. *S. cerevisiae* has a strong fermentative power and rapid establishment capacity, allowing it to outcompete native yeasts and contaminating microorganisms. Rapid establishment limits the proliferation of unwanted microorganisms and reduces the risk of organoleptic deviation after alcoholic fermentation. *S. cerevisiae* rapidly consumes oxygen in the wort, thus limiting oxidation and the growth of contaminating aerobic microorganisms. By quickly occupying the environment and limiting contamination, *S. cerevisiae* makes it possible to reduce the use of sulfites upon receipt of the grape musts. Moreover, this work shows that the production of the killer toxin Sc54Kt could maximize the bioprotective effect of the strain Sc54, allowing winemakers to reduce or suppress the use of sulfites during winemaking. Industrial-scale trials are now necessary to confirm the value of this strain as a bioprotectant.

## Figures and Tables

**Table 1:** List of killer *S. cerevisiae* strains utilized, with their source of isolation. Killing activity screening against two strains of *B. bruxellensis* (B1 and B250) and one *H. uvarum* (Hu3137) on YPD-MB agar. Legends: ++ strong killing effect (diameter of halo 10–13 mm); + apparent killing effect (diameter of halo between 8 and 9 mm); +/− mild killing effect (diameter of halo < 8 mm and not consistent); − no killing effect; Nd: activity not determined.

*S. cerevisiae* Codes	Killing Activity Agar Medium		
	B1	B250	Hu3137
Sc54	++	++	++
S342	++	+	+
S340	+	+	+/−
S334	+	−	+/−
VL2	++	+	Nd

**Table 2:** Growth parameters of *B. bruxellensis* and *H. uvarum* in single culture and co-culture with *S. cerevisiae* Sc54 in synthetic must SM300 at 22 °C.

Sensitive Strain	Killer Strain	µmax (h^−1^)	Final Population (CFU/mL) (9 Days)
B1 ^1^	Control	0.126 ± 0.001 ^a^	2.72 × 10^8^ ± 0.11 × 10^8 a^
	Sc54 ^2^	0.042 ± 0.003 ^b^	8.10 × 10^4^ ± 0.82 × 10^4 b^
B250 ^1^	Control	0.123 ± 0.002 ^a^	2.7 × 10^8^ ± 0.06 × 10^8 a^
	Sc54 ^2^	0.026 ± 0.01 ^c^	3.44 × 10^4^ ± 0.87 × 10^4 b^
Hu3137 ^1^	Control	0.204 ± 0.001 ^a^	5.8 × 10^7^ ± 1.2 × 10^7 a^
	Sc54 ^2^	−0.01 ± 0.012 ^b^	0 ^b^

^1^ initial concentration 10^4^ CFU/mL, ^2^ initial concentration 10^6^ CFU/mL, Letter (a, b, c) corresponds to statistical groups (post hoc “Bonferroni”, *p* < 0.05) obtained by the pairwise comparison of values between B1, B250, and Hu3137 single culture and co-culture with the bioprotectant strains at initial concentration 10^4^ and 10^6^ CFU/mL, respectively.

**Table 3:** Evaluation of the killing activity of two concentrations of Sc54Kt, 0.185 and 0.5 mg/mL, on agar.

Killer Toxin	Halo Diameter (mm)					
	*H. uvarum* Hu3137		*B. bruxellensis* B1		*B. bruxellensis* B250	
	0.5 mg/mL	0.185 mg/mL	0.5 mg/mL	0.185 mg/mL	0.5 mg/mL	0.185 mg/mL
Sc54Kt	13	7	11	5	10	5

Purification procedures outlined in the Materials and Methods. One arbitrary unit (AU) corresponds to the toxin concentration needed to produce 1 mm inhibition zone around the well.

## Data Availability

The original contributions presented in this study are included in the article. Further inquiries can be directed to the corresponding author.

## References

[1] Alexandre H., Puyo M., Tourdot-Maréchal R. (2023). Bioprotection in Winemaking. New Advances in Saccharomyces [Working Title].

[2] Escribano-Viana R., González-Arenzana L., Garijo P., Fernández L., López R., Santamaría P., Gutiérrez A.R. (2022). Bioprotective Effect of a *Torulaspora delbrueckii/Lachancea thermotolerans*-Mixed Inoculum in Red Winemaking. Fermentation.

[3] Rubio-Bretón P., Gonzalo-Diago A., Iribarren M., Garde-Cerdán T., Pérez-Álvarez E.P. (2018). Bioprotection as a Tool to Free Additives Winemaking: Effect on Sensorial, Anthocyanic and Aromatic Profile of Young Red Wines. LWT.

[4] Tzamourani A., Evangelou A., Ntourtoglou G., Lytra G., Paraskevopoulos I., Dimopoulou M. (2024). Effect of *Non-Saccharomyces* Species Monocultures on Alcoholic Fermentation Behavior and Aromatic Profile of Assyrtiko Wine. Appl. Sci..

[5] Napa-Almeyda C.A., Criado C., Mayta-Hancco J., Silva-Jaimes M., Condezo-Hoyos L., Pozo-Bayón M.Á. (2023). *Non-Saccharomyces* Yeast Strains, Aromatic Compounds and Sensory Analysis of Italy and Negra Criolla Pisco from the Moquegua Region of Peru. Fermentation.

[6] Yao M., Wang F., Arpentin G. (2023). Bioprotection as a Tool to Produce Natural Wine: Impact on Physicochemical and Sensory Analysis. BIO Web Conf..

[7] SantˈAna A.S., Lemos Junior W.J.F. (2024). Microbial Synergies and Their Impact on Economic and Quality Innovation in Sustainable Winemaking: Yeast and Lactic Acid Bacteria Interconnections. Food Biosci..

[8] Liu S., Zhao Y., Li Y., Lou Y., Feng X., Yang B. (2023). Comparison of Phenolic Profiles of Albino Bilberry (*Vaccinium Myrtillus* L.) Wines Fermented by *non-Saccharomyces* Yeasts. Food Biosci..

[9] Bordet F., Joran A., Klein G., Roullier-Gall C., Alexandre H. (2020). Yeast–Yeast Interactions: Mechanisms, Methodologies and Impact on Composition. Microorganisms.

[10] Bartowsky E.J., Henschke P.A. (2004). The ‘Buttery’ Attribute of Wine—Diacetyl—Desirability, Spoilage and Beyond. Int. J. Food Microbiol..

[11] Liu S. (2003). Practical Implications of Lactate and Pyruvate Metabolism by Lactic Acid Bacteria in Food and Beverage Fermentations. Int. J. Food Microbiol..

[12] Barbe J.-C., Pelonnier-Magimel E., Windhotz S., Masneuf Pomarède I. (2020). Sensory Characterisation of Wines without Added Sulfites via Specific and Adapted Sensory Profile. OENO One.

[13] Simonin S., Honoré-Chedozeau C., Monnin L., David-Vaizant V., Bach B., Alexandre H., Chatelet B., Tourdot-Marechal R. (2022). Bioprotection on Chardonnay Grape: Limits and Impacts of Settling Parameters. Aust. J. Grape Wine Res..

[14] Windholtz S., Dutilh L., Lucas M., Maupeu J., Vallet-Courbin A., Farris L., Coulon J., Masneuf-Pomarède I. (2021). Population Dynamics and Yeast Diversity in Early Winemaking Stages without Sulfites Revealed by Three Complementary Approaches. Appl. Sci..

[15] Reiter T., Montpetit R., Byer S., Frias I., Leon E., Viano R., Mcloughlin M., Halligan T., Hernandez D., Figueroa-Balderas R. (2021). Transcriptomics Provides a Genetic Signature of Vineyard Site and Offers Insight into Vintage-Independent Inoculated Fermentation Outcomes. mSystems.

[16] Windholtz S., Redon P., Lacampagne S., Farris L., Lytra G., Cameleyre M., Barbe J.-C., Coulon J., Thibon C., Masneuf-Pomarède I. (2021). *Non-Saccharomyces* Yeasts as Bioprotection in the Composition of Red Wine and in the Reduction of Sulfur Dioxide. LWT.

[17] Hamm D., Muñoz González B., Morata A., Loira I., González C., Escott C. (2023). Use of Other Species in Winemaking, and Their Interaction with *Saccharomyces cerevisiae*. New Advances in Saccharomyces.

[18] Comuzzo P., Del Fresno J.M., Voce S., Loira I., Morata A. (2023). Emerging Biotechnologies and Non-Thermal Technologies for Winemaking in a Context of Global Warming. Front. Microbiol..

[19] Giménez P., Just-Borras A., Pons P., Gombau J., Heras J.M., Sieczkowski N., Canals J.M., Zamora F. (2023). Biotechnological Tools for Reducing the Use of Sulfur Dioxide in White Grape Must and Preventing Enzymatic Browning: Glutathione; Inactivated Dry Yeasts Rich in Glutathione; and Bioprotection with *Metschnikowia pulcherrima*. Eur. Food Res. Technol..

[20] Puyo M., Simonin S., Klein G., David-Vaizant V., Quijada-Morín N., Alexandre H., Tourdot-Maréchal R. (2023). Use of Oenological Tannins to Protect the Colour of Rosé Wine in a Bioprotection Strategy with *Metschnikowia pulcherrima*. Foods.

[21] Viola E., Naselli V., Prestianni R., Pirrone A., Porrello A., Amato F., Savastano R., Maggio A., Carusi M., Seminerio V. (2025). The Impact of a *Saccharomyces cerevisiae* Bio-Protective Strain during Cold Static Clarification on Catarratto Wine. AIMS Microbiol..

[22] Wang C., Mas A., Esteve-Zarzoso B. (2016). The Interaction between *Saccharomyces cerevisiae* and non-*Saccharomyces* Yeast during Alcoholic Fermentation Is Species and Strain Specific. Front. Microbiol..

[23] El Dana F., Hayar S., Colosio M.-C. (2021). Selection of Three Indigenous Lebanese Yeast *Saccharomyces cerevisiae* with Physiological Traits from Grape Varieties in Western Semi-Desert and Pedoclimatic Conditions in the Bekaa Valley. Fermentation.

[24] Esteve-Zarzoso B., Belloch C., Uruburu F., Querol A. (1999). Identification of Yeasts by RFLP Analysis of the 5.8S rRNA Gene and the Two Ribosomal Internal Transcribed Spacers. Int. J. Syst. Evol. Microbiol..

[25] Lopes C.A., Sangorrín M.P. (2010). Optimization of Killer Assays for Yeast Selection Protocols. Rev. Argent. Microbiol..

[26] Harju S., Fedosyuk H., Peterson K.R. (2004). Rapid Isolation of Yeast Genomic DNA: Bust n’ Grab. BMC Biotechnol..

[27] Legras J.-L., Karst F. (2003). Optimisation of Interdelta Analysis for *Saccharomyces cerevisiae* Strain Characterisation. FEMS Microbiol. Lett..

[28] De Celis M., Ruiz J., Martín-Santamaría M., Alonso A., Marquina D., Navascués E., Gómez-Flechoso M.Á., Belda I., Santos A. (2019). Diversity of *Saccharomyces cerevisiae* Yeasts Associated to Spontaneous and Inoculated Fermenting Grapes from Spanish Vineyards. Lett. Appl. Microbiol..

[29] Cappello M.S., Bleve G., Grieco F., Dellaglio F., Zacheo G. (2004). Characterization of *Saccharomyces cerevisiae* Strains Isolated from Must of Grape Grown in Experimental Vineyard. J. Appl. Microbiol..

[30] Belloch C., Barrio E., García M.D., Querol A. (1998). Phylogenetic Reconstruction of the Yeast Genus *Kluyveromyces*: Restriction Map Analysis of the 5.8S rRNA Gene and the Two Ribosomal Internal Transcribed Spacers. Syst. Appl. Microbiol..

[31] Bely M., Sablayrolles J.-M., Barre P. (1990). Automatic Detection of Assimilable Nitrogen Deficiencies during Alcoholic Fermentation in Oenological Conditions. J. Ferment. Bioeng..

[32] Evers M.S., Roullier-Gall C., Morge C., Sparrow C., Gobert A., Vichi S., Alexandre H. (2023). Thiamine and Biotin: Relevance in the Production of Volatile and Non-Volatile Compounds during *Saccharomyces cerevisiae* Alcoholic Fermentation in Synthetic Grape Must. Foods.

[33] Puyo M., Mas P., Roullier-Gall C., Romanet R., Lebleux M., Klein G., Alexandre H., Tourdot-Maréchal R. (2023). Bioprotection Efficiency of *Metschnikowia* Strains in Synthetic Must: Comparative Study and Metabolomic Investigation of the Mechanisms Involved. Foods.

[34] Branco P., Francisco D., Chambon C., Hébraud M., Arneborg N., Almeida M.G., Caldeira J., Albergaria H. (2014). Identification of Novel GAPDH-Derived Antimicrobial Peptides Secreted by *Saccharomyces cerevisiae* and Involved in Wine Microbial Interactions. Appl. Microbiol. Biotechnol..

[35] Santos A., San Mauro M., Bravo E., Marquina D. (2009). PMKT2, a New Killer Toxin from *Pichia membranifaciens*, and Its Promising Biotechnological Properties for Control of the Spoilage Yeast *Brettanomyces bruxellensis*. Microbiology.

[36] Büyüksırıt-Bedir T., Kuleaşan H. (2022). Purification and Characterization of a *Metschnikowia pulcherrima* Killer Toxin with Antagonistic Activity against Pathogenic Microorganisms. Arch. Microbiol..

[37] Büyüksırıt Bedir T., Kuleaşan H. (2021). A Natural Approach, the Use of Killer Toxin Produced by *Metschnikowia pulcherrima* in Fresh Ground Beef Patties for Shelf Life Extention. Int. J. Food Microbiol..

[38] Bradford M.M. (1976). A Rapid and Sensitive Method for the Quantitation of Microgram Quantities of Protein Utilizing the Principle of Protein-Dye Binding. Anal. Biochem..

[39] Kielkopf C.L., Bauer W., Urbatsch I.L. (2020). Bradford Assay for Determining Protein Concentration. Cold Spring Harb. Protoc..

[40] Kielkopf C.L., Bauer W., Urbatsch I.L. (2020). Methods for Measuring the Concentrations of Proteins. Cold Spring Harb. Protoc..

[41] Kumar Sharma P., Chand D. (2012). Purification and Characterization of Thermostable Cellulase Free Xylanase from *Pseudomonas* Sp. XPB-6. Adv. Microbiol..

[42] Carboni G., Fancello F., Zara G., Zara S., Ruiu L., Marova I., Pinna G., Budroni M., Mannazzu I. (2020). Production of a Lyophilized Ready-to-Use Yeast Killer Toxin with Possible Applications in the Wine and Food Industries. Int. J. Food Microbiol..

[43] Comitini F., Ingeniis De J., Pepe L., Mannazzu I., Ciani M. (2004). *Pichia anomala* and *Kluyveromyces wickerhamii* Killer Toxins as New Tools against *Dekkera/Brettanomyces* Spoilage Yeasts. FEMS Microbiol. Lett..

[44] Romano P., Suzzi G., Comi G., Zironi R. (1992). Higher Alcohol and Acetic Acid Production by Apiculate Wine Yeasts. J. Appl. Bacteriol..

[45] Aragno J., Fernandez-Valle P., Thiriet A., Grondin C., Legras J.-L., Camarasa C., Bloem A. (2024). Two-Stage Screening of *Metschnikowia spp*. Bioprotective Properties: From Grape Juice to Fermented Must by *Saccharomyces cerevisiae*. Microorganisms.

[46] Li Y.-Q., Hu K., Xu Y.-H., Mei W.-C., Tao Y.-S. (2020). Biomass Suppression of *Hanseniaspora uvarum* by Killer *Saccharomyces cerevisiae* Highly Increased Fruity Esters in Mixed Culture Fermentation. LWT.

[47] Hu K., Zhao H., Edwards N., Peyer L., Tao Y., Arneborg N. (2022). The Effects of Cell-Cell Contact between *Pichia kluyveri* and *Saccharomyces cerevisiae* on Amino Acids and Volatiles in Mixed Culture Alcoholic Fermentations. Food Microbiol..

[48] Branco P., Sabir F., Diniz M., Carvalho L., Albergaria H., Prista C. (2019). Biocontrol of *Brettanomyces/Dekkera bruxellensis* in Alcoholic Fermentations Using Saccharomycin-Overproducing *Saccharomyces cerevisiae* Strains. Appl. Microbiol. Biotechnol..

[49] Tristezza M., Tufariello M., Capozzi V., Spano G., Mita G., Grieco F. (2016). The Oenological Potential of *Hanseniaspora uvarum* in Simultaneous and Sequential Co-Fermentation with *Saccharomyces cerevisiae* for Industrial Wine Production. Front. Microbiol..

[50] Vaquero C., Escott C., Heras J.M., Carrau F., Morata A. (2022). Co-Inoculations of *Lachancea thermotolerans* with Different *Hanseniaspora* spp.: Acidification, Aroma, Biocompatibility, and Effects of Nutrients in Wine. Food Res. Int..

[51] Cibrario A., Miot-Sertier C., Paulin M., Bullier B., Riquier L., Perello M.-C., De Revel G., Albertin W., Masneuf-Pomarède I., Ballestra P. (2020). *Brettanomyces bruxellensis* Phenotypic Diversity, Tolerance to Wine Stress and Wine Spoilage Ability. Food Microbiol..

[52] Kemsawasd V., Viana T., Ardö Y., Arneborg N. (2015). Influence of Nitrogen Sources on Growth and Fermentation Performance of Different Wine Yeast Species during Alcoholic Fermentation. Appl. Microbiol. Biotechnol..

[53] Albergaria H., Arneborg N. (2016). Dominance of *Saccharomyces cerevisiae* in Alcoholic Fermentation Processes: Role of Physiological Fitness and Microbial Interactions. Appl. Microbiol. Biotechnol..

[54] Branco P., Coutinho R., Malfeito-Ferreira M., Prista C., Albergaria H. (2021). Wine Spoilage Control: Impact of Saccharomycin on *Brettanomyces bruxellensis* and Its Conjugated Effect with Sulfur Dioxide. Microorganisms.

[55] Huang M., Liu X., Li X., Sheng X., Li T., Tang W., Yu Z., Wang Y. (2022). Effect of *Hanseniaspora uvarum*–*Saccharomyces cerevisiae* Mixed Fermentation on Aroma Characteristics of Rosa Roxburghii Tratt, Blueberry, and Plum Wines. Molecules.

[56] Wang C., Mas A., Esteve-Zarzoso B. (2015). Interaction between *Hanseniaspora uvarum* and *Saccharomyces cerevisiae* during Alcoholic Fermentation. Int. J. Food Microbiol..

[57] Li L., Yuan C., Zhang L., Chu R., Yu Q., Cai J., Yang T., Zhang M. (2024). The Impact of Simultaneous Inoculation with *Torulaspora delbrueckii* and *Hanseniaspora uvarum* Combined with *Saccharomyces cerevisiae* on Chemical and Sensory Quality of Sauvignon Blanc Wines. Front. Microbiol..

[58] Comitini F., Agarbati A., Canonico L., Galli E., Ciani M. (2020). Purification and Characterization of WA18, a New Mycocin Produced by *Wickerhamomyces anomalus* Active in Wine Against *Brettanomyces bruxellensis* Spoilage Yeasts. Microorganisms.

[59] Villalba M.L., Mazzucco M.B., Lopes C.A., Ganga M.A., Sangorrín M.P. (2020). Purification and Characterization of *Saccharomyces eubayanus* Killer Toxin: Biocontrol Effectiveness against Wine Spoilage Yeasts. Int. J. Food Microbiol..

[60] De Ullivarri M.F., Mendoza L.M., Raya R.R. (2014). Killer Activity of *Saccharomyces cerevisiae* Strains: Partial Characterization and Strategies to Improve the Biocontrol Efficacy in Winemaking. Antonie Van. Leeuwenhoek.

[61] Branco P., Francisco D., Monteiro M., Almeida M.G., Caldeira J., Arneborg N., Prista C., Albergaria H. (2017). Antimicrobial Properties and Death-Inducing Mechanisms of Saccharomycin, a Biocide Secreted by *Saccharomyces cerevisiae*. Appl. Microbiol. Biotechnol..

[62] Albergaria H., Francisco D., Gori K., Arneborg N., Gírio F. (2010). *Saccharomyces cerevisiae* CCMI 885 Secretes Peptides That Inhibit the Growth of Some *non-Saccharomyces* Wine-Related Strains. Appl. Microbiol. Biotechnol..

[63] Pereznevado F., Albergaria H., Hogg T., Girio F. (2006). Cellular Death of Two *non-Saccharomyces* Wine-Related Yeasts during Mixed Fermentations with *Saccharomyces cerevisiae*. Int. J. Food Microbiol..

[64] Comitini F., Mannazzu I., Ciani M. (2009). *Tetrapisispora phaffii* Killer Toxin Is a Highly Specific β-Glucanase That Disrupts the Integrity of the Yeast Cell Wall. Microb. Cell Factories.

[65] Bussey H., Sherman D., Somers J.M. (1973). Action of Yeast Killer Factor: A Resistant Mutant with Sensitive Spheroplasts. J. Bacteriol..

[66] Kurzweilová H., Sigler K. (1993). Factors Affecting the Susceptibility of Sensitive Yeast Cells to Killer Toxin K1. Folia Microbiol..

[67] Breinig F., Tipper D.J., Schmitt M.J. (2002). Kre1p, the Plasma Membrane Receptor for the Yeast K1 Viral Toxin. Cell.

[68] Boone C., Sommer S.S., Hensel A., Bussey H. (1990). Yeast KRE Genes Provide Evidence for a Pathway of Cell Wall Beta-Glucan Assembly. J. Cell Biol..

[69] De La Peña P., Barros F., Gascón S., Lazo P.S., Ramos S. (1981). Effect of Yeast Killer Toxin on Sensitive Cells of *Saccharomyces cerevisiae*. J. Biol. Chem..

[70] Skipper N., Bussey H. (1977). Mode of Action of Yeast Toxins: Energy Requirement for *Saccharomyces cerevisiae* Killer Toxin. J. Bacteriol..

[71] Dignard D., Whiteway M., Germain D., Tessier D., Thomas D.Y. (1991). Expression in Yeast of a cDNA Copy of the K2 Killer Toxin Gene. Molec. Gen. Genet..

[72] Lukša J., Podoliankaitė M., Vepštaitė I., Strazdaitė-Žielienė Ž., Urbonavičius J., Servienė E. (2015). Yeast β-1,6-Glucan Is a Primary Target for the *Saccharomyces cerevisiae* K2 Toxin. Eukaryot. Cell.

[73] Novotna D., Flegelova H., Janderova B. (2004). Different Action of Killer Toxins K1 and K2 on the Plasma Membrane and the Cell Wall of *Saccharomyces cerevisiae*. FEMS Yeast Res..

[74] Flegelová H., Novotná D., Vojtíšková K., Janderová B. (2002). Isolation and Characterization of *Saccharomyces cerevisiae* Mutants with a Different Degree of Resistance to Killer Toxins K1 and K2. FEMS Yeast Res..

[75] Schmitt M.J. (1995). Cloning and Expression of a cDNA Copy of the Viral K28 Killer Toxin Gene in Yeast. Molec. Gen. Genet..

[76] Rodríguez-Cousiño N., Maqueda M., Ambrona J., Zamora E., Esteban R., Ramírez M. (2011). A New Wine *Saccharomyces cerevisiae* Killer Toxin (Klus), Encoded by a Double-Stranded RNA Virus, with Broad Antifungal Activity Is Evolutionarily Related to a Chromosomal Host Gene. Appl. Environ. Microbiol..

